# Distinct temporal brain dynamics in bipolar disorder and schizophrenia during emotion regulation

**DOI:** 10.1017/S0033291719000217

**Published:** 2020-02

**Authors:** Liwen Zhang, Hui Ai, Esther M. Opmeer, Jan-Bernard C. Marsman, Lisette van der Meer, Henricus G. Ruhé, André Aleman, Marie-José van Tol

**Affiliations:** 1 Department of Pharmacology, National University of Singapore, Singapore; 2 Shenzhen Key Laboratory of Affective and Social Neuroscience, Shenzhen University, Shenzhen, China; 3 University of Groningen, University Medical Center Groningen, Cognitive Neuroscience Center, Groningen, the Netherlands; 4 Department of Rehabilitation, Lentis Psychiatric Institute, Zuidlaren, the Netherlands; 5 Department of Psychiatry, Radboud University Medical Center, Nijmegen, Netherlands; 6 Department of Psychology, University of Groningen, Groningen, the Netherlands

**Keywords:** Bipolar disorder, cognitive reappraisal, emotion regulation, finite impulse response modeling, prefrontal cortex, schizophrenia

## Abstract

**Background:**

Disturbances in emotion regulation (ER) are characteristic of both patients with bipolar disorder (BD) and schizophrenia (SZ). We investigated the temporal dynamics of brain activation during cognitive ER in BD and SZ to understand the contribution of temporal characteristics of disturbed ER to their unique and shared symptomatology.

**Method:**

Forty-six participants performed an ER-task (BD, *n* = 15; SZ, *n* = 16; controls, *n* = 15) during functional magnetic resonance imaging, in which they were instructed to use cognitive reappraisal techniques to regulate their emotional responses. Finite impulse response modeling was applied to estimate the temporal dynamics of brain responses during cognitive reappraisal (*v.* passive attending) of negative pictures. Group, time, and group × time effects were tested using multivariate modeling.

**Results:**

We observed a group × time interaction during ER in the ventrolateral prefrontal cortex (VLPFC), supplementary motor area (SMA) and inferior occipital gyrus. Patients with SZ demonstrated initial hyper-activation of the VLPFC and SMA activation that was not sustained in later regulatory phases. Response profiles in the inferior occipital gyrus in SZ showed abnormal activation in the later phases of regulation. BD-patients showed general blunted responsivity in these regions.

**Conclusions:**

These results suggest that ER-disturbances in SZ are characterized by an inefficient initialization and failure to sustain regulatory control, whereas in BD, a failure to recruit regulatory resources may represent initial deficits in formulating adequate representations of the regulatory needs. This may help to further understand how ER-disturbances give rise to symptomatology of BD and SZ.

## Introduction

Inadequate emotion regulation (ER) poses a threat to personal well-being (Gross, 2002) and is a central part of various psychiatric disorders, including bipolar disorder (BD) (Rowland *et al*., 2013; Wolkenstein *et al*., 2014) and schizophrenia (SZ) (Livingstone *et al*., 2009; van der Meer *et al*., 2009, 2014). Neural abnormalities during ER have been observed in both BD and SZ (Morris *et al*., 2012; Townsend *et al*., 2013; van der Meer *et al*., 2014; Kanske *et al*., 2015), implying a shared pathology that might relate to the occurrence of psychotic symptoms (Modinos *et al*., 2010), which are also present in 20–50% of BD-patients (Keck *et al*., 2003). On the other hand, BD and SZ are characterized by unique symptomatology, with strong fluctuations in affect during depression and mania in BD, and a predominantly flat affect in SZ (Phillips *et al*., 2003*b*). A direct comparison between BD and SZ indeed suggested a differential neural profile during cognitive ER, with BD-patients showing increased prefrontal involvement compared to SZ-patients (Morris *et al*., 2012). In this and other studies, lower lateral prefrontal recruitment was also observed in SZ-patients compared to controls (Morris *et al*., 2012; van der Meer *et al*., 2014), whereas BD has been associated with both lower (Townsend *et al*., 2013) and higher (Morris *et al*., 2012) lateral prefrontal activation during ER compared to controls. Of note, it has been demonstrated in healthy individuals that ER areas do not show continuous and stable but instead sequential, orchestrated involvement across time during reappraisal (Goldin *et al*., 2008). However, in most ER-studies to date, the temporal variations of brain responses during regulatory attempts occurring over long periods of time were not taken into account. This raises the question whether abnormalities in SZ arise from a failure to engage frontal regulatory areas *per se* or from a failure to sustain initial frontal engagement during the full duration of ER. Similarly, it is unknown whether altered frontal recruitment is an early, late, or constant characteristic of BD. However, elucidating the temporal dynamics of the complex response underlying ER is essential to understand the contributions of emotion dysregulation to the unique symptomatology of both disorders.

The most investigated strategy of cognitive ER is cognitive reappraisal, aiming at influencing the emotional intensity and connotation of a stimulus by reinterpreting its meaning (Gross, 2001, 2002; Gross and John, 2003). Given that reappraisal is relevant for the occurrence and treatment of psychosis (Kuipers *et al*., 2006) that is prevalent in both SZ and BD, the current study focused on reappraisal. In healthy individuals, reappraisal relies on adequate interactions between the prefrontal cortex [PFC, including the ventrolateral PFC (VLPFC), dorsolateral PFC (DLPFC), and dorsomedial PFC (DMPFC)] and limbic affective areas (including the amygdala and ventral striatum) (Ochsner *et al*., 2012; Buhle *et al*., 2014; Morawetz *et al*., [Bibr ref31][Bibr ref31], [Bibr ref32][Bibr ref32]). Interestingly, activation of the lateral and medial PFC was shown to be at maximum during an early phase (approximately 4.5 s after ER-onset), followed by attenuation of the amygdala response at a late stage (approximately 10.5 s after ER-onset) (Goldin *et al*., 2008). This is consistent with the idea that the role of the VLPFC during regulation is to receive information about the affective value of the stimulus from the limbic areas, and forward this information to the DLPFC to call for prefrontal control over the limbic areas (Kohn *et al*., 2014). Moreover, this indicates that essential information for understanding the complex psychopathology related to ER-deficiencies may be missed when averaging the blood-oxygen-level dependent (BOLD) signal over long periods of time, as is often done in functional magnetic resonance imaging (fMRI) analysis. However, no study has investigated the temporal dynamics of ER in patients with BD and SZ yet.

We therefore aim to investigate the temporal dynamics underlying reappraisal in BD and SZ, using finite impulse response (FIR) modeling to estimate the timing and shape of the BOLD responses. Given the distinctive emotional symptoms seen in BD and SZ, we hypothesized differential temporal patterns within prefrontal and limbic areas between these two disorders. Moreover, healthy individuals were included to investigate shared abnormalities during ER in BD and SZ.

## Materials and methods

### Participants

Patients (23 BD-patients, 51 SZ-patients) were recruited through mental health care centers in the context of a study investigating insight (van der Meer *et al*., 2013). Because illness insight is strongly associated with psychosis (David, 1999) and emotional processing (van der Werf-Eldering *et al*., 2011), we only included patients with good insight and matched BD and SZ with insight scores for the current analysis, leading to a sample of 20 SZ-patients and 21 BD-patients. Also, 17 healthy controls (HC) were included. For patients, the following inclusion criteria were applied: (1) no change in medication at least 1 week before scanning; (2) no electroconvulsive therapy in the year prior to scanning; (3) no psychiatric disorders other than BD or SZ and no comorbidity with other psychiatric disorders (e.g. substance use disorder); (4) no somatic/neurological disorders with known influence on brain functioning. Because patients were recruited in the context of studying insight in psychosis, a history of psychotic symptoms was required for patients with BD. HC were required to have no current or past psychiatric disorders. Presence of MRI-incompatibilities and somatic or neurological disorder affecting the central nervous system was an exclusion criterion for all participants.

This study was approved by the Medical Ethical Committee of the University Medical Center Groningen and was performed according to the Helsinki Declaration (2008). All participants provided written informed consent.

### Clinical assessments

To confirm the diagnosis of BD (including a history of psychotic symptoms) or SZ, and to exclude life-time psychiatric disorders in HC, the Mini International Neuropsychiatric Interview-Plus 5.0.0 (MINI-Plus; Sheehan *et al*., 1998) was administered. For all participants, severity of current depressive symptoms was measured with the Quick Inventory of Depressive Symptomatology (QIDS; Rush *et al*., 2003), intelligence was estimated using the Dutch Adult Reading Test (DART; Schmand *et al*., 1991), and daily preferred ER-strategy was assessed with the Emotion Regulation Questionnaire (ERQ; Gross and John, 2003). Only in patients, severity of current positive and negative symptoms was measured with the Positive and Negative Syndrome Scale (PANSS; Kay *et al*., 1987), severity of current mania with the Young Mania Rating Scale (YMRS; Young *et al*., 1978), and clinical and cognitive insight with the Schedule of Assessment of Insight-Expanded version (SAI-E; Kemp and David, 1997), and the Beck Cognitive Insight Scale (BCIS; Beck *et al*., 2004) respectively.

### Emotion regulation task

**Fig. 1.**
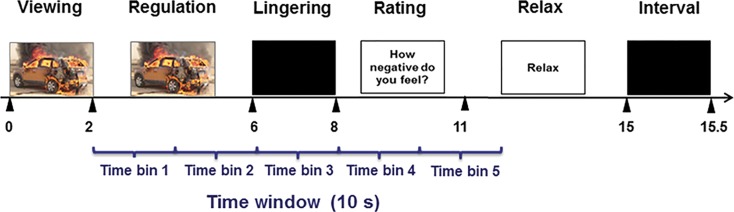
Illustration of the task procedure during a reappraise trial and the time window used for FIR modeling. During attend-negative trials, participants were instructed to continue to attend to the picture during 4 s following the cue and preceding lingering. The black triangles indicate the start/end of each task phase. FIR, finite impulse response.

The ER-task has been described in detail in a previous ER-study (van der Meer *et al*., 2014). Briefly, 88 negative and 22 neutral pictures were selected from the International Affective Picture System (Lang *et al*., 2005; van der Meer *et al*., 2014). In each trial, a neutral or negative picture was shown with the instruction to view (2 s). Afterward, participants were asked to either reappraise or attend to this picture for 4 s, leading to three conditions: *attend-neutral*, *attend-negative* (viewing the picture without changing the experienced emotion) and *reappraise-negative* (*reinterpreting to decrease the negative emotional intensity*). Following the picture, a black screen was presented (lingering; 2 s), followed by affective rating on a four-point scale (1 – not negative at all; 4 – extremely negative) (3 s). Subsequently, a screen with the word ‘relax’ cued participants to relax (4 s). Finally, a black screen appeared to signal the next trial (0.5 s) (Fig. 1). The whole experiment was split into two runs, with a rest period in between during which the anatomical scan was acquired. See online Supplement for task details. Within runs, conditions were presented pseudorandomized, with equal distribution of conditions over blocks of 9 or 10 trials. In between blocks, a fixation-cross of 20 s was presented.

Although the task also included a *suppress* (i.e. inhibit emotional expressions) and an *increase* (i.e. effortfully increase the intensity of negative emotions) condition, we focused on reappraisal in the current study (see online Supplement for the results in relation to suppress and increase).

### Data acquisition

fMRI-data were collected using a 3.0 Tesla scanner (Philips Intera, Best, NL) at the University Medical Center Groningen, equipped with an 8-channel SENSE head-coil. Using a T2-weighted echo planar imaging sequence, 37 axial slices were acquired per volume in an ascending, interleaved order (slice-thickness = 3.5 mm, slice-gap = 0 mm; TR = 2.0 s; TE = 30 ms; flip-angle = 70°; in-plane resolution 64 × 62 pixels; voxel size of 3.5 × 3.5 mm). Additionally, a T1-weighted 3D anatomical image (170 slices; TR = 9 ms; TE = 3.54 ms; voxel size of 1 × 1 × 1 mm; flip-angle = 8°) was acquired.

### Statistical analysis

#### Clinical variables and behavioral data

Demographics, psychometric assessments, and behavioral data were analyzed in SPSS (v.22.0), using one-way analysis of variances (ANOVAs), *t* tests, and χ^2^ tests where appropriate.

A repeated-measures ANOVA was performed to analyze the ratings during the task, with condition (3; *attend-neutral*, *attend-negative*, *reappraise*) as within-subjects factor and group (3; HC, SZ, BD) as between-subjects factor. Significance level was set to *p* < 0.05 (two-tailed) for all analyses.

#### fMRI data pre-processing

fMRI-data were pre-processed with statistical parametric mapping [SPM12b (v5970); Wellcome Trust Centre for Neuroimaging, London, UK] in Matlab 7.8.0 (R2009a; Mathworks, Natick, MA, USA). Both functional and anatomical images were manually reoriented to the anterior–posterior commissure plane before pre-processing. Pre-processing consisted of slice-time correction, realignment, co-registration of the functional images to the anatomical image, normalization to Montreal Neurological Institute (MNI) (2 × 2 × 2 mm) space and smoothing with a full-width at half-maximum Gaussian kernel of 8 mm.

#### Finite impulse response modeling

To quantify the shape/timing of the hemodynamic response (HR) during reappraisal, a general linear model with a FIR basis set was built to estimate condition-specific time-courses without *a priori* assumptions on their shape. Notably, this FIR-modeling does not take into consideration the known delay of the HR-function. Also, given the late-cueing paradigm, activation in response to negative pictures during the initial viewing phase was assumed to be comparable between conditions, especially since pictures under different conditions were matched on valence, arousal, and complexity. Therefore, to estimate ER-relevant signals and to avoid including activation related to the last part of the previous trial, a 10 s-time-window was set following the start of the regulation instruction, which was not fixed to the TR (Fig. 1). The 10 s-time-window was modeled with five triangular basis functions (‘tent function’), each of which spanned 2 s. This way, activation in response to both viewing and regulating were included within our time window of interest when assuming the known delay in HR function peak response of approximately 5 s. We however started modeling at the onset of the cue to either regulate or attend, as differences related to effortful regulation compared to passive attend were our primary interest.

In each participant's first-level model, regressors (with five tents) were included per condition (*attend-neutral*, *attend-negative*, *reappraise*, *increase* and *suppress*). FIR-estimates of the HR were calculated for each time bin (5) and condition (5) per participant. To investigate the temporal characteristics specific to reappraisal, we specified the contrast *reappraise* > *attend-negative* per time-bin at the first level and these contrasts were then entered in our main analysis (see below: 3dMVM modeling). To check whether effects were driven by reappraising or attending negative-pictures, we repeated our analyses for the contrast *attend-negative* > *attend-neutral*.

Whole-brain multivariate modeling (3dMVM in AFNI) was used to examine the temporal dynamics during *reappraise* > *attend-negative*, with group (3; HC, BD, SZ) as between-subjects factor, and time (5; 1, 2, 3, 4, 5) as within-subjects factor. 3dMVM allows quantification of the inter-correlation among the within-subject variables, which is likely high for the consecutive time-bins (Chen *et al*., 2014, 2015), and has proven to be effective at detecting subtle shape changes of HR (Chen *et al*., 2015). Since our goal was to identify temporal differences between groups over the regulation period and to be able to detect commonalities in abnormal temporal patterns between SZ and BD as compared to HC, we tested for group (3) × time (5) interactions across all time bins, and for main effects of group and time. Because we were additionally interested in the differences in the temporal profile between patients with BD and SZ, we additionally tested for group (2) × time (5) interactions for BD *v.* SZ. Furthermore, to check their deviations from normal across the regulatory period, we explored BD and SZ *v.* HC. Notably, all above-mentioned contrasts were defined in one 3dMVM model, and group differences were not examined for each time bin separately. We repeated our analyses after adding QIDS score in the model including all three groups and PANSS score (total scores) in the model including BD- and SZ-patients as covariates of no interest, respectively. In addition, the possible influence of medication was explored by adding dummy variables coding for usage of medication types [i.e. antipsychotics (yes/no), anti-depressants (yes/no), mood-stabilizers (yes/no), benzodiazepines (yes/no), with no medication as the implicit option when all other dummies were coded ‘no’ (i.e. 0)] in the model including patients only.

For all analyses, Monte Carlo simulations were used to correct for multiple comparisons (i.e. 3d ClustSim in AFNI v18.1.04 updated on April 2018) (Cox *et al*., 2016). All results were thresholded at a voxel-wise threshold of *p* < 0.001 uncorrected, and should meet minimum cluster extent criteria (*k* > 74 for the main three group model and *k* > 70 for the models in which we controlled for medication and PANSS within patients) to hold a family-wise error (FWE) correction of *p* < 0.05. See online Supplemental material for details of cluster extent definition.

## Results

### Sample characteristics

Due to incomplete imaging data, excessive head-movements (i.e. >3 mm/3° in any direction) or missing Eprime log-files, 11 cases were excluded (BD: *n* = 5; SZ: *n* = 4; HC: *n* = 2). To match groups on age, another BD-patient was excluded. For the final analyses, 15 HC, 15 BD-patients, and 16 SZ-patients were included. One HC missed questionnaires/behavioral data and was excluded from behavioral analyses but included in the MRI analyses. Notably, a part of our sample (12 SZ-patients and 12 HC) was included in a previous study (van der Meer *et al*., 2014), without FIR-analysis being applied.

All groups were comparable on age, sex, level of education, intelligence and ERQ-scores, but differed on QIDS-score (HC < BD < SZ; *p*s < 0.01). Moreover, BD and SZ were comparable on YMRS and insight scores (i.e. SAI-E/BCIS; *p*s > 0.55), but SZ showed higher PANSS-scores than BD (*p* = 0.048). See Table 1 and online Supplementary Table S2 for details.

**Table 1.** Demographic and clinical characteristics in all groups

		HC (*N* = 15)	SZ (*N* = 16)	BD (*N* = 15)	Likelihood ratio	*F*	*t*^a^	*p*
Age	*M* (s.d.)	33.60 (11.1)	31.75 (8.7)	39.87 (12.5)	–	2.35	–	0.11 (0.04^a^)
Level of education	*M* (s.d.)	5.60 (0.9)	5.56 (1.0)	6.07 (0.7)	–	1.50	–	0.24 (0.12^a^)
Gender (male/female)	*N*	10/5	12/4	6/9	4.30	–	–	0.12
BD type (I/II)	*N*			13/2				
ERQ^b^_reappraisal	*M* (s.d.)	4.87 (1.0)	4.57 (1.4)	4.21 (1.5)	–	1.01	–	0.37
ERQ^b^_suppression	*M* (s.d.)	2.66 (1.2)	3.67 (0.9)	3.38 (1.5)	–	2.35	–	0.12
DART n correct	*M* (s.d.)	40.93 (6.6)	38.00 (6.5)	42.73 (3.9)	–	2.64	–	0.08 (0.03^a^)
QIDS-SR^c^	*M* (s.d.)	2.00 (1.2)	9.13 (4.5)	5.27 (5.4)	–	11.04		<0.001* (0.01^a^)
Depressive state^c^ (yes/no)	*N*		5/11	2/13				
YMRS^c^	*M* (s.d.)	–	1.75 (1.7)	1.40 (1.5)	–	–	0.60	0.55
Manic state^c^ (yes/no)	*N*		0/16	0/15				
SAI_E scores	*M* (s.d.)	–	21.30 (2.5)	22.27 (2.1)	–	–	0.60	0.55
BCIS_self certainty	*M* (s.d.)	–	7.56 (3.1)	7.4 (2.5)	–	–	0.16	0.88
BCIS_reflectiveness	*M* (s.d.)	–	15.25 (4.0)	14.73 (4.0)	–	–	0.36	0.72
BCIS_composite	*M* (s.d.)	–	7.69 (4.7)	7.33 (4.7)	–	–	0.21	0.84
PANSS_positive	M (s.d.)	–	12.38 (4.8)	9.47(2.7)	–	–	2.06	0.048*
PANSS_negative	*M* (s.d.)	–	14.31 (4.8)	8.93 (2.3)	–	–	4.01	0.001*
PANSS_general	*M* (s.d.)	–	26.69 (7.1)	21.53(3.6)	–	–	2.53	0.02*

BCIS, Beck cognitive insight scale; DART, Dutch Reading Test for Adults; ERQ, emotion regulation questionnaire; *M*, mean; PANSS, Positive and Negative Syndrome Scale; QIDS, Quick Inventory of Depressive Symptomatology; SAI_E, Schedule of Assessment of Insight-Expanded version; s.d., standard error; YMRS, Young Mania Rating Scale.

a Comparison between two patients groups (BD and SZ).

b The ERQ consists of ten items: six items measure the cognitive reappraisal strategy and four items the suppression strategy. Because of the unequal number of items between the two subscales, the total score for each subscale was divided by the number of items per subscale.

c We defined a depressive state with a QIDS score of >10 (http://www.ids-qids.org/) and a manic state with a cut-off score of 8 on the YMRS (Mercer and Becerra, 2013).

* *p* < 0.05.

### Behavioral results

Across all participants, there was a main effect of condition on emotion ratings (*F*_(2,84)_ = 132.38, *p* < 0.01; see online Supplementary Fig. S1 for ratings in all conditions). *Post-hoc t* tests indicated most negative ratings after attend-negative (*M* = 2.63, s.e.m. = 0.08) and lowest after attend-neutral (*M* = 1.20, s.e.m. = 0.03), with the ratings following reappraisal in between (*M* = 2.23, s.e.m. = 0.09) (*attend-negative* < *reappraise-negative* < *attend-neutral*; *p*s < 0.001). No significant main effect of group (*F*_(2,42)_ = 2.70, *p* = 0.08) and interaction of group × condition were found (*F*_(4,84)_ = 0.337, *p* = 0.85).

### FIR modeling results during reappraise > attend-negative

#### Comparisons between SZ, BD, and HC

**Fig. 2.**
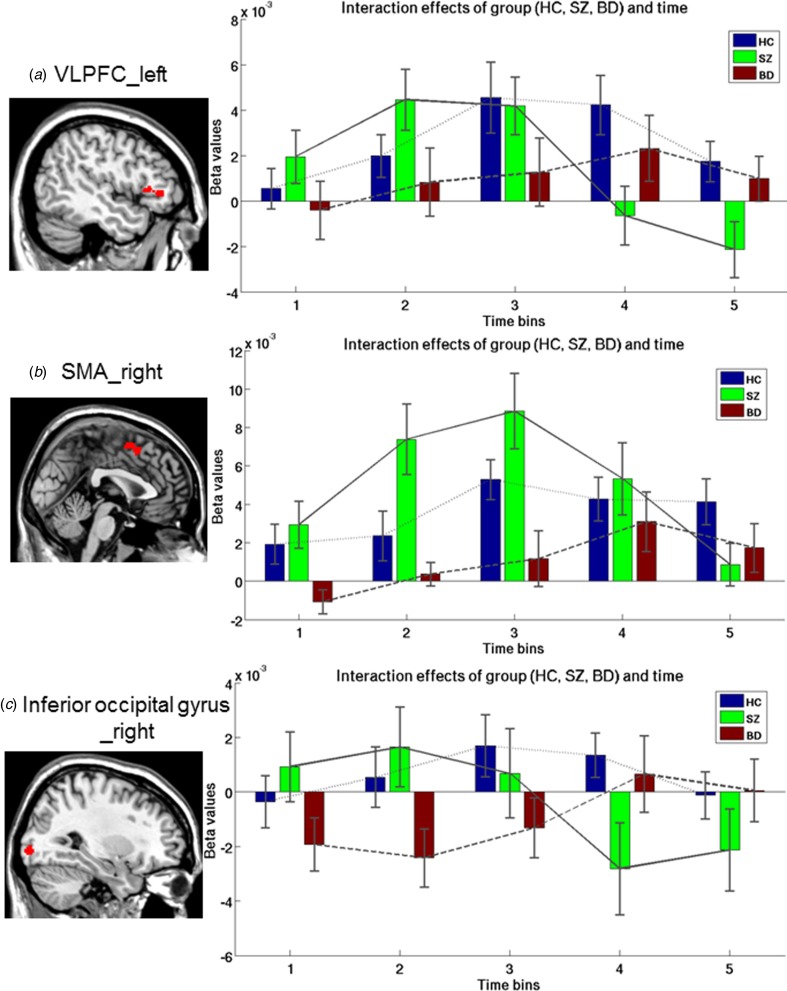
Interaction effects between group and time in HC, patients with BD, and SZ at the contrast reappraise > attend-negative. Effects were observed (*a*) in the VLPFC, (*b*) SMA, and (*c*) inferior occipital gyrus. Time bins start at the presentation of regulation cue (i.e. time bin 1, 0–2 s post instruction). The presented time course is averaged over significant voxels. The line connecting the mean responses in each time bin visualizes the temporal response for each group (BD, dashed dark gray; HC, dashed light gray; SZ, straight dark gray). BD, bipolar disorder; HC, healthy controls; SZ, schizophrenia; SMA, supplementary motor area; VLPFC, ventrolateral prefrontal cortex.

Reappraisal-sensitive temporal profiles are illustrated in Table 2 and Fig. 2. A main effect of time was shown in the VLPFC, DLPFC, DMPFC, temporal pole, middle temporal gyrus (MTG), STG, and supplementary motor area (SMA). No main effect of group was observed, but a group × time interaction on reappraisal > attend-negative was found in the left VLPFC, right inferior occipital gyrus, and right SMA. Plotting of the time-courses showed that SZ had normal to hyper-engagement of the VLPFC and inferior occipital gyrus in the early phases (time bin 1–3), with a drop in activation during the later phases (time bin 4–5), where HC showed more sustained higher activation in the later time bins when comparing reappraise-negative to attend-negative (Fig. 2*a*, *c*). For the SMA, SZ showed hyper-activation in the early phases (time bin 2–3) relative to HC, followed by a gradual decrease toward normal at time bin 4 and hypoactivation at time bin 5 (Fig. 2*b*) when contrasting reappraise-negative to attend-negative. BD failed to show heightened responses in the VLPFC and SMA in the early phases relative to HC, but showed some normalization of responses in the later time bins (time bin 4–5; Fig. 2*a*, *b*). In the inferior occipital gyrus, BD was characterized by blunted response relative to HC (Fig. 2*c*). Similar results were obtained after controlling for QIDS (online Supplementary Table S3). Observed findings in relation to reappraisal could not be attributed to the group difference over time during the attend-negative (online Supplementary Fig. S3). See online Supplementary Fig. S6 for plots including the viewing phase preceding the reappraise or attend cue.

**Table 2.** Group comparisons during reappraise > attend negative (cluster extent threshold *k* > 74)

Contrasts	*K*	BA	Side	MNI coordinates			Peak intensity
				*x*	*y*	*z*	
BD *v.* SZ *v.* HC Main effect of time							
VLPFC	1044	47	L	−44	28	−8	10.70
Dorsolateral prefrontal cortex	340	46	L	−28	58	22	9.65
Dorsomedial prefrontal cortex	312	24		0	26	40	8.09
Temporal pole	82	38	L	−50	10	−20	7.10
Temporal pole	203	38	R	50	20	−14	10.67
MTG	292	21	L	−56	−32	−2	14.49
Superior temporal gyrus	369	39	L	−54	−60	20	11.49
SMA	832	6	L	−2	4	66	21.79
Interaction: Group (BD, SZ, HC) × Time							
VLPFC	83	45	L	−48	34	2	4.35
Inferior occipital gyrus	111	18	R	28	−92	−2	6.29
SMA	126	6	R	2	14	50	4.54
BD *v.* HC Main effect of group							
MTG	81	21	R	58	6	−22	25.03
Dorsolateral prefrontal cortex /VLPFC	297	46/44	L	−30	16	35	26.80
SZ *v.* HC Main effect of group							
Inferior occipital gyrus	186	19	R	36	−90	−4	22.85
VLPFC	102	45	L	−52	28	14	20.91
Interaction: Group (SZ, HC) × Time							
VLPFC	90	45	R	40	34	16	13.64

Significance at *P*_FWE_ < 0.05 with a height threshold of *p* < 0.001.

#### Comparisons between BD and SZ

Planned comparisons between BD and SZ revealed no main effect of group or group × time effects. Controlling for PANSS scores and medication types did not change this observation (online Supplementary Tables 4 and 6).

#### Comparisons between SZ and BD to HC

To objectify deviations from normal, we compared SZ-patients and BD-patients to HC separately. The comparison between SZ and HC showed a main effect of group in the left VLPFC extending toward the DLPFC, and right inferior occipital gyrus, resulting from lower activation in SZ than HC (Table 2). Results of group × time interaction effects showed that SZ demonstrated an abnormal response over time in the right VLPFC, with early abnormal recruitment in time bin 2 (online Supplementary Fig. 2). In the model comparing BD to HC, a main effect of group was observed in the left DLPFC extending to the VLPFC and right MTG, with lower activation in BD compared to HC. No group × time effect was observed.

No effects of group × time were observed for the conditions *suppress* and *increase*. See online Supplement for post-hoc explorations.

## Discussion

In this study, we investigated for the first time the temporal dynamics of cognitive ER in patients with BD, SZ, and HC. We demonstrated that BD and SZ were characterized by distinct temporal profiles of engaging brain regions during reappraisal compared to attending negative pictures, with unique deviations from normal in the VLPFC, SMA, and inferior occipital gyrus. Of those regions, both the VLPFC and SMA have been consistently indicated as part of an ER-network (Ochsner *et al*., 2012; Kohn *et al*., 2014). In short, SZ-patients showed initial normal- to hyper-recruitment of the VLPFC, but failed to sustain this activation in late phases of regulation, together with early hyperactivation and non-sustained activation of the SMA in the late phase of activation. BD-patients showed blunted responses in these areas, especially during early regulation phases. This suggests that inefficient initialization and a failure to sustain regulatory control may underlie ER-abnormalities in SZ, whereas a failure to engage regulatory areas may underlie ER-abnormalities in BD. These disorder-specific temporal fingerprints may help understand how abnormalities in reappraisal contribute to the unique symptomatology in different psychiatric disorders.

In SZ, consistent with previous findings (Morris *et al*., 2012; van der Meer *et al*., 2014), we observed lower activation in the VLPFC during reappraisal compared to HC. Notably, our results add to previous findings that although hypo-activation was lower in general in the pars triangular part of the VLPFC bordering the DLPFC, activation in a more ventral part of the triangular part of the VLPFC was characterized by a failure to sustain activation following a subtle initial over-recruitment. The VLPFC is a key component of the neural circuitry involved in appraisals that initiates controlled ER (Dixon *et al*., 2017), and has been linked to response selection and inhibition during reappraisal (Ochsner and Gross, 2005; Ochsner *et al*., 2012; Morawetz *et al*., 2016), inhibition of self-perspective during theory of mind tasks (van der Meer *et al*., 2011), top-down control over emotional information (Phillips *et al*., 2003*a*, 2008), detection of salience to signal the need for control (Kohn *et al*., 2014), and self-reflection (van der Meer *et al*., 2010, 2013; Murray *et al*., 2012). This suggests that the VLPFC is an important area for forming a meta-cognitive representation of emotional information and for detecting a need for control during reappraisal. Based on the suggested roles of the VLPFC in ER, we propose that in SZ, the salience of the emotional information is initially processed but in a possibly inefficient way as indicated by the early over-recruitment of this area compared with HC. This suggestion is in line with the emotion paradox in SZ that they have shown reduced emotional expression (flat affect), but with normal or even enhanced emotional experience compared to healthy individuals (Aleman and Kahn, 2005; Kring and Moran, 2008; Cohen and Minor, 2010). Also, this suggests that the emotional information is processed for further elaboration, but that SZ-patients fail to keep an adequate emotional reappraisal online, indicated by the non-sustained VLPFC activation, that may hinder controlled ER (Dixon *et al*., 2017). This resulting suboptimal reappraisal may be associated with a higher negative affect (compared to HC) based on the depression severity scores, and might be investigated further in relation to inappropriate affect featured in SZ.

In BD on the other hand, we observed a stable blunted response of a cluster encompassing the VLPFC extending to the DLPFC, and in the MTG. The DLPFC is important for maintaining reappraisal goals in working memory during ER (Wager *et al*., 2004; Ochsner *et al*., 2012) and for explicit reasoning (Ochsner and Gross, 2005; Kohn *et al*., 2014). The MTG has been proposed to be important for ER success (Morawetz *et al*., 2017*b*) and to play an important intermediate role together with the DLPFC and amygdala during ER related to cognitive control over emotions, similar to the VLPFC (Ochsner *et al*., 2012). In line with this previous reasoning, the blunted VLPFC/DLPFC and temporal response pattern may indicate that abnormal ER in BD results from a general failure to form an adequate reappraisal of the emotion situation and associated need for regulatory cognitive control.

The suggested dissociation between BD and SZ (as compared to HC) based on adequate recognition of saliency and associated need for control resources in relation to VLPFC functioning is further supported by the observed differential responses in the SMA and the inferior occipital gyrus extending to the middle occipital gyrus. In comparison with HC, we observed hyper-recruitment of the SMA in SZ in the early phases of regulation, whereas BD showed blunted SMA activation during the entire regulation window. The SMA has been suggested to be an essential intermediate unit between the prefrontal areas and limbic affective areas during ER (Kohn *et al*., 2014). Specifically, the SMA has been involved in stimulus reconceptualization (Kohn *et al*., 2014) and cognitive demands (Urry *et al*., 2009) during ER. Therefore, the over-recruitment of the SMA in the early phase of regulation together with low recruitment in the late phase may indicate a counterproductive call upon cognitive control. As the hyperrecruitment of the SMA was most pronounced at the second time bin, and the BOLD response usually peaks five seconds post-event, this may suggest that this hyperrecruitment might be related to the initial viewing phase and thus may represent abnormalities related to stimulus attribution. This is supported by online Supplemental Fig. S6. In BD, blunted VLPFC response during reappraisal was accompanied by blunted responses of the SMA compared to HC, suggesting that these regions are recruited to a less extent, which may reflect a diminished call for adequate cognitive regulatory control. Though less well known, the inferior occipital gyrus has been implicated as an important region for ER in mood disorders (Pico-Perez *et al*., 2017) and emotion recognition (Wiggins *et al*., 2016). Near-normal early recruitment of the inferior occipital gyrus in SZ may therefore represent initial correct recognition of negative emotional information which seemed not sustained in later phases (time bin 4–5). In BD, the blunted pattern of response in the inferior occipital gyrus, VLPFC and SMA, implies a general failure to identify regulatory needs. We therefore propose that ER-deficits in SZ are primarily driven by an inefficient initialization and failure to sustain regulatory control, while ER-deficits in BD are characterized by a primary failure to index regulatory needs. Though testing for group × time × instruction was beyond the scope of this paper, we explored post-hoc whether similar group differences were observed during the increase and suppress conditions. Plotting of the time courses in the regions resulting from the main analyses indicated similar direction of response differences between groups during increasing negative emotions, but not during suppression (see online Supplement). However, no group or group × time effect during either increase (>attend-negative) or suppress (>attend-negative) reached statistical significance. Though clearly in need of further study, these differential temporal patterns of activation in BD and SZ in the VLPFC, SMA and inferior occipital gyrus may be specific to effortful downregulation of negative emotional material, and not for intensifying or suppressing emotional experience.

Some limitations need to be mentioned. First, most patients were taking medication and potential confounding effects could not be excluded. However, after controlling for medication, comparable results between patient groups were observed (online Supplementary Tables 2 and 6). Second, our sample size was modest, which increases the risk of inflated effect size estimates (*Ioannidis*, 2005). However, we believe that our observations are important to report as they result from highly selective and difficult to recruit patient populations. Comparability between patient groups was improved by selecting BD-patients with a history of psychosis and SZ-patients with comparable levels of illness insight as the BD-patients. Third, BD- and SZ-patients differed on current depression severity and positive/negative symptoms, but did so mainly with mild forms of symptomatology. The temporal dynamics of reappraisal during more severe mood states remain unclear, since differential neural correlates between depressed and euthymic states have been shown in BD (Rive *et al*., 2015). Unfortunately, our small sample size of mainly euthymic/non-symptomatic patients prevented us from exploring these factors. Also, we could not control for the heterogeneity within the BD group, related to characteristics of the most recent episode and bipolar type I *v.* bipolar type II. Replication in a bigger (medication-free) sample with better matching of clinical variables and during different polarity states is recommended before strong conclusions can be drawn. Forth, although results of group × time interaction during *reappraise* > *attend-negative* indicate that the observed cortical temporal responses were specific to reappraisal compared to attend-negative, we did not find an association between PFC temporal-responses and negative affect ratings in an exploratory correlation analysis (see online Supplement). A narrow rating range (four-point scale) might have confounded this result. Furthermore, we did not have affect ratings of the participants in response to the same stimuli before regulation. Therefore, our calculation method of ER success (i.e. mean rating after *attend-negative* > mean rating after *reappraisal*) may not reflect regulation success sufficiently, which also prevented us from exploring dynamics of regulation success. Fifth, there was no effect of reappraisal on amygdala activation between the attend and regulate condition, which might be related to the late-cueing design (Ochsner *et al*., 2012) and that the time window following the regulation instruction was too short to allow the amygdala to reach its maximum response (Goldin *et al*., 2008). Finally, reappraisal has shown differential modulating effects for positive and negative stimuli (Rive *et al*., 2015), while we only focused on negative stimuli. Including positive conditions in future work may further clarify the emotional disturbances in BD (e.g. problems with down-regulating positive affect during mania) and SZ (e.g. difficulty in up-regulating positive affect).

In conclusion, our findings of differential temporal responses between BD and SZ during reappraisal may indicate that SZ-patients predominantly show an inefficient initialization of regulatory attempts followed by a failure of sustaining activation in areas important for signaling reappraisal needs, whereas BD may have a more general deficiency in initiating reappraisal due to suboptimal recognition of the saliency of the stimuli and detecting the need for regulation. These differences between BD and SZ might be of potential interest as disorder-specific biomarkers and may enable a better understanding of these two disorders by linking the observed differential temporal dynamics to distinct emotional symptomatology of BD (e.g. switching between emotional poles) and SZ (e.g. inappropriate affect) in the future.

## Supplementary material

For supplementary material accompanying this paper visit http://dx.doi.org/10.1017/S0033291719000217. click here to view supplementary material
