# Supplementary material for: Distinct temporal brain dynamics in bipolar disorder and schizophrenia during emotion regulation
Source: Psychol Med. 2019 Feb 18;50(3):413–21. doi: 10.1017/S0033291719000217 (PMC7025159; doi:10.1017/S0033291719000217)
Supplement: Supplementary file 1 [file S0033291719000217sup001.doc]

# Distinct temporal brain dynamics in bipolar disorder and schizophrenia during emotion regulation

Liwen Zhang*, Hui Ai*, Esther M. Opmeer, Jan-Bernard C. Marsman, Lisette van der Meer,

Henricus G. Ruhé, André Aleman, and Marie-José van Tol^♥^

^♥^Corresponding author.

*These authors contributed equally to this study.

Correspondence address: University of Groningen, University Medical Center Groningen, Antonius Deusinglaan 2, 9713 AW, Groningen, The Netherlands.

Fax: +31 050 363 8875. Tel: +31 050 361 6405.

E-mail addresses: m.j.van.tol@umcg.nl

# Supplemental Materials and methods

Inclusion and exclusion criteria

For patients, the following inclusion criteria were applied: 1) no change in medication at least one week before scanning; 2) no electroconvulsive therapy in the year prior to scanning; 3) no psychiatric disorders other than bipolar disorder or schizophrenia and no comorbidity with other psychiatric disorders (e.g., substance use disorder); 4) no somatic/neurological disorders with known influence on brain functioning. Because patients were recruited in the context of studying insight in psychosis, a history of psychotic symptoms was required for patients with bipolar disorder. Healthy controls were required to have no current or past psychiatric disorders. Presence of MRI-incompatibilities and somatic or neurological disorder affecting the central nervous system was an exclusion criterion for all participants.

## Emotion regulation task

The ER-task has been described in detail and shown valid in a previous ER-study (van der Meer et al. 2014). In short, 88 negative and 22 neutral pictures were selected from the International Affective Picture System (Lang et al. 2005; van der Meer et al. 2014). Extremely negative pictures were not selected to avoid potential psychotic experiences. In each trial, a neutral or negative picture was shown with the instruction to view (2s). Afterwards, participants were asked to either reappraise or attend to this picture for 4s, leading to three conditions: attend-neutral, attend-negative and reappraise-negative. Following the picture, a black screen was presented (lingering; 2s), followed by affective rating on a 4-point scale (1-not negative at all; 4-extremely negative) (3s). Subsequently, a screen with the word “relax” cued participants to relax (4s). Finally, a black screen appeared to signal the next trial (0.5s) (Figure 1).

During attend-trials (22 negative and 22 neutral pictures), participants were instructed to continue viewing the picture without changing the experienced emotion. During reappraise-trials, participants were instructed to reinterpret negative pictures (n=22) to decrease the negative emotional intensity. Valence and arousal levels of negative pictures were matched between the attend-negative and reappraise conditions. Additionally, the task also included a suppress (i.e., inhibit emotional expressions) and an increase (i.e., effortfully increase the intensity of negative emotions via reinterpretation) condition. Prior to scanning, participants were trained on applying the experimental instructions. During this training session, participants had to tell the experimenter how they applied the regulation strategy in order for the researcher to check whether they understood the task. After the fMRI scanning, participants were asked whether they had been able to regulate emotions as instructed.

The whole experiment was split into two runs, with a rest period in between during which the anatomical scan was acquired. Within runs, conditions were presented pseudorandomized, with equal distribution of conditions over blocks of 9 or 10 trials. In between blocks, a fixation-cross of 20s was presented.

Steps to define cluster extent threshold

Firstly, we calculated the square-root of the ResMS.nii image of each participant; secondly, we calculated the acf parameters on the square-rooted residual files via 3dFWHMx in the latest version of AFNI (version 18.1.04); thirdly, we calculated an average of each acf value over subjects and used these values as input for 3dClustSim; finally, we read out the NN1 2-sided results for holding a voxel-threshold of *p*<.001 and cluster-corrected threshold of *p*<.05. Given the MonteCarlo Simulation generates extents with small variations, we ran it for 100 times to calculate the average cluster extent. This procedure resulted in defining one cluster extent threshold for three group 3dMVM models (i.e., k>74) and one cluster extent threshold for the models covarying medication and PANSS within patients (i.e. k>70).

# Supplemental data analysis

## *Data analysis of main task effects*

Analysis was conducted with statistical parametric mapping (SPM12b [v5970]; Wellcome Trust Centre for Neuroimaging, London, UK) implemented in Matlab 7.8.0 (R2009a; Mathworks, Natick, MA, USA). In order to check the task validity by testing the average prefrontal activation across time bins during reappraise > attend negative in all participants, the contrast images were entered into a one-sample t-test model. Threshold was set at *p*<0.05 (FWE corrected) on cluster-level, with an initial voxel-defining threshold of *p*<0.001.

Statistical analysis of exploration of correlation with reappraisal success

To investigate whether prefrontal activation differences during reappraisal represented regulation success, activation in clusters in the prefrontal cortex showing a group×time interaction during reappraise > attend-negative (i.e., the left ventrolateral prefrontal cortex and right dorsolateral prefrontal cortex) were correlated to regulation success. Regulation success was calculated as the difference score between the mean rating score after attend-negative and mean rating score after reappraisal (attend-negative>reappraise). Correlation analyses were conducted per time bin in all participants, with a threshold of *p*<0.05 (two-tailed).

Data analysis for the contrast reappraise > baseline and attend negative > baseline

## We investigated whether the main effect from reappraise > attend negative was related to changes in attend (> baseline) or reappraise (>baseline) by examining the time courses from these two contrasts within the activated clusters from our main analysis (reappraise > attend negative: VLPFC and SMA). The baseline was the un-modeled fixation period as well as other un-modeled parts of the task in the FIR model.

## **Supplemental results**

## Results of main task effects

During reappraisal, average activation across time bins was observed in the dorsolateral and ventral lateral prefrontal cortex, dorsomedial prefrontal cortex, superior frontal gyrus, middle frontal gyrus, mid-cingulate cortex, angular gyrus, middle temporal gyrus, supplementary motor area, and thalamus (Table S1). There were no regions with higher activation during *attend-negative* compared to *reappraise*.

## Results of correlation analyses

There were no correlations between the left ventrolateral prefrontal cortex (VLPFC) and the regulation success for each time bin in all participants [Time bin 1: *r*_VLPFC_=0.09, *p*=0.54; Time bin 2: *r*_VLPFC_=0.03, *p*=0.87; Time bin 3: *r*_VLPFC_=-0.04, *p*=0.79; Time bin 4: *r*_VLPFC_=-0.16, *p*=0.31; Time bin 5: *r*_VLPFC_=0.03, *p*=0.82].

Results of reappraise > baseline and attend negative > baseline

We observed a significant interaction between group and time in the left VLPFC for reappraise (>baseline) but not for attend negative (>baseline) (Results are listed in Supplementary Table 5). Therefore, the effect observed in the left VLPFC was not contributed by attend negative.

Regarding SMA, an interaction effect between group and time was observed at a subthreshold level (*p*=0.1) during reappraise (>baseline) but no effect was observed during attend negative (>baseline).

Results of suppress > attend negative

There were no group or group×time interaction effects in the areas from our main analysis (i.e. VLPFC, SMA and inferior occipital gyrus) for suppress (> attend negative). See Supplementary figure 4 for plotting of the signal across time bins in the three groups.

Results of increase > attend negative

Similar trends of activation differences among groups as reappraise > attend negative in areas from our main analyses were observed from plots during increase > attend negative (Supplementary Figure 5), but without showing significant group or group×time interaction effects. By visual inspection, there was recruitment of the VLPFC, SMA and the inferior occipital gyrus in SZ that dropped in later time bins whereas HC showed more sustained activation during the later time bins. BD did not have heightened recruitment in the VLPFC, SMA and inferior occipital gyrus, but showed some recruitment during late time bins, to a lesser extent than HC.

Supplementary Tables

**Supplementary Table 1 Main task effects during *reappraise > attend negative* in all participants across time bins**

| Region | Side | BA | Cluster size | T | MNI  coordinates | | |
| --- | --- | --- | --- | --- | --- | --- | --- |
|  |  |  |  |  | x | y | z |
| Dorsolateral prefrontal cortex  /ventrolateral prefrontal cortex | L | 44/45/47 | 1833 | 6.92  6.65  6.37 | -42  -52  -42 | 16  24  28 | 38  0  -10 |
| Ventrolateral prefrontal cortex  /temporal pole | R | 38/45/47 | 498 | 4.73  4.68  4.65 | 54  44  50 | 26  4  18 | -4  -32  -14 |
| Dorsomedial prefrontal gyrus  /supplementary motor area  /mid-cingulate cortex | L | 6/8/32 | 980 | 6.05  5.60  4.97 | -4  -2  10 | 2  14  10 | 64  60  62 |
| Superior frontal gyrus/  Middle frontal gyrus | L | 10 | 230 | 6.42 | -22 | 48 | 22 |
| Middle temporal gyrus  /angular gyrus | L | 21/39 | 1300 | 4.96  4.88  4.80 | -60  -54  -56 | -32  -58  -48 | 2  16  -2 |
| Thalamus |  |  | 185 | 5.71  4.64  3.40 | 12  -8  0 | -10  -14  -18 | 0  2  -4 |

**Supplementary Table 2 Medication use in patient groups**

|  |  | SZ | BD | Likelihood ratio | *p* |
| --- | --- | --- | --- | --- | --- |
| Psychopharmacological medication |  |  |  |  |  |
| *Antipsychotics* | N | 15 | 6 | 10.24 | <.05 |
| Quetiapine | N | 2 | 4 | 1.01 | .39 |
| Olanzapine | N | 5 | 2 | 1.46 | .23 |
| Risperidone | N | 1 | 0 | 1.35 | .25 |
| Aripiprazole | N | 6 | 0 | 9.29 | <.05 |
| Clozapine | N | 2 | 0 | 2.78 | .10 |
| *Benzodiazepine* | N | 3 | 0 | 4.27 | .04 |
| *Antidepressants* | N | 9 | 3 | 8.43 | <.05 |
| Trazodone | N | 0 | 1 | 1.48 | .22 |
| Bupropion | N | 0 | 1 | 1.48 | .22 |
| Mirtazapine | N | 1 | 0 | 1.35 | .25 |
| SSRIs | N | 5 | 1 | 3.24 | .07 |
| Venlafaxine | N | 3 | 0 | 4.27 | .04 |
| *Mood stablizer* | N | 0 | 11 | 22.93 | <.05 |
| Valproic acid | N | 0 | 4 | 6.44 | .01 |
| Lithium | N | 0 | 11 | 22.93 | <.05 |
| *None* | N | 1 | 1 | .01 | 1 |

Abbreviation: SSRIs=selective serotonin reuptake inhibitors.

# Supplementary Table 3 Group x Time interaction in BD, SZ and HC after including QIDS as a covariate (Cluster extent threshold k > 74)

| Contrasts | K | BA | Side | MNI coordinates  x y z | | | | | | | Peak intensity |
| --- | --- | --- | --- | --- | --- | --- | --- | --- | --- | --- | --- |
| **Bipolar disorder versus schizophrenia versus healthy controls**  **Main effect of time** | | | | | | | | | | | |
| Ventrolateral prefrontal cortex | 1023 | 47 | L | | -44 | | 28 | | -8 | | 10.70 |
| Dorsolateral prefrontal cortex | 336 | 46 | L | | -28 | | 58 | | 22 | | 9.65 |
| Dorsomedial prefrontal cortex | 302 | 24 |  | | 0 | | 26 | | 40 | | 8.09 |
| Temporal pole | 202 | 38 | R | | 50 | | 20 | | -14 | | 10.67 |
| Middle temporal gyrus | 279 | 21 | L | | -56 | | -32 | | -2 | | 14.49 |
| Superior temporal gyrus | 365 | 39 | L | | -54 | | -60 | | 20 | | 11.49 |
| Supplementary motor area | 786 | 6 | L | | -2 | | 4 | | 66 | | 21.79 |
| **Interaction: Group (bipolar disorder, schizophrenia, healthy controls) × Time** | | | | | | | | | | | |
| Ventrolateral prefrontal cortex | 79 | 45 | L | | -48 | | 34 | | 2 | | 4.35 |
| Inferior occipital gyrus | 112 | 18 | R | | 28 | | -92 | | -2 | | 6.29 |
| Supplementary motor area | 126 | 6 | R | | 2 | | 14 | | 50 | | 4.54 |
| **Bipolar disorder versus schizophrenia** | | | | | | | | | | | |
| **Interaction: Group (bipolar disorder, schizophrenia) × Time** | | | | | | | | | | | |
| Dorsolateral prefrontal cortex | 72 | 46 | R | | 26 | | 54 | | 18 | | 10.25 |
| **Bipolar disorder versus healthy controls**  **Main effect of group** | | | | | | | | | | | |
| Middle temporal gyrus | 81 | 21 | R | 58 | | 6 | | -22 | | 25.03 | |
| Dorsolateral prefrontal cortex  /Ventrolateral prefrontal cortex | 297 | 46/44 | L | -30 | | 16 | | 35 | | 26.80 | |
| **Schizophrenia versus healthy controls**  **Main effect of group** | | | | | | | | | | | |
| Inferior occipital gyrus | 186 | 19 | R | 36 | | -90 | | -4 | | 22.85 | |
| Ventrolateral prefrontal cortex | 102 | 45 | L | -52 | | 28 | | 14 | | 20.91 | |
| **Interaction: Group (schizophrenia, healthy controls) × Time** | | | | | | | | | | | |
| Ventrolateral prefrontal cortex | 90 | 45 | R | 40 | | 34 | | 16 | | 13.64 | |

# Supplementary Table 4 Group x Time interaction in BD and SZ after including PANSS total scores as a covariate (corrected for the voxel-wise level *p*<.001, but uncorrected for cluster extent; cluster extent threshold of k > 70)

| Contrasts | K | BA | Side | MNI coordinates  x y z | | | | Peak intensity |
| --- | --- | --- | --- | --- | --- | --- | --- | --- |
| **Bipolar disorder versus schizophrenia** | | | | | | | | |
| **Interaction: Group (bipolar disorder, schizophrenia) × Time** | | | | | | | | |
| Ventrolateral prefrontal cortex | 80 | 45 | L | | -48 | 34 | 2 | 6.70* |
| Supplementary motor area | 161 | 6 | R | | 2 | 14 | 50 | 7.78* |

*Indicated significance at P_FWE_<.05 with a height threshold of *p*<.001

**Supplementary Table 5** Effects for the contrast reappraise > baseline

| Contrasts | K | BA | Side | MNI coordinates  x y z | | | | Peak intensity |
| --- | --- | --- | --- | --- | --- | --- | --- | --- |
| **Bipolar disorder versus schizophrenia versus healthy controls**  **Interaction: Group (bipolar disorder, schizophrenia, healthy controls) × Time** | | | | | | | | |
| Ventrolateral prefrontal cortex | 76 | 47 | L | | -46 | 30 | -6 | 7.95 |
| Supplementary motor area | 12 | 6 | R | | 2 | 14 | 50 | 3.07 |

**Supplementary Table 6** Group x Time interaction in BD and SZ after including medication types as covariates (corrected for the voxel-wise level p<.001, p_FWE_=.05, k>70)

| Contrasts | K | BA | Side | MNI coordinates  x y z | | | | Peak intensity |
| --- | --- | --- | --- | --- | --- | --- | --- | --- |
| **Bipolar disorder versus schizophrenia** | | | | | | | | |
| **Interaction: Group (bipolar disorder, schizophrenia) × Time** | | | | | | | | |
| Ventrolateral prefrontal cortex | 80 | 45 | L | | -48 | 30 | -4 | 6.70* |
| Supplementary motor area | 161 | 6 | R | | 2 | 14 | 50 | 7.78* |

*Indicated significance at P_FWE_<.05 with a height threshold of *p*<.001

**Supplementary Figure**


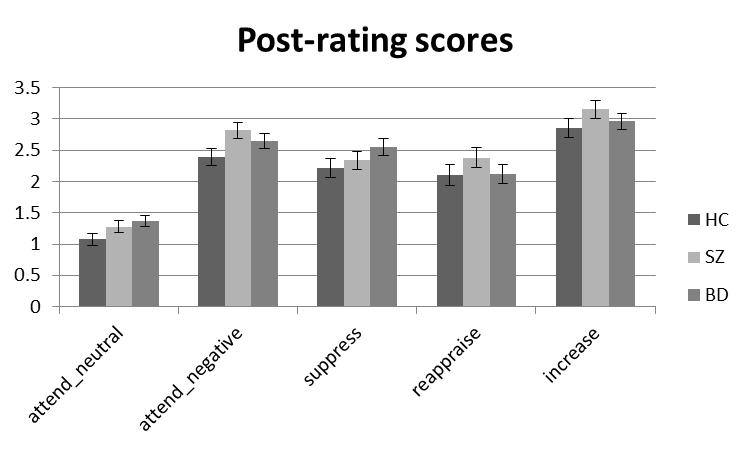


**Supplementary Figure 1** Results of rating score for the conditions attend-negative, attend-neutral, reappraise, suppress and increase in healthy controls, bipolar group and schizophrenia group. One error bar represents one standard error in each direction.


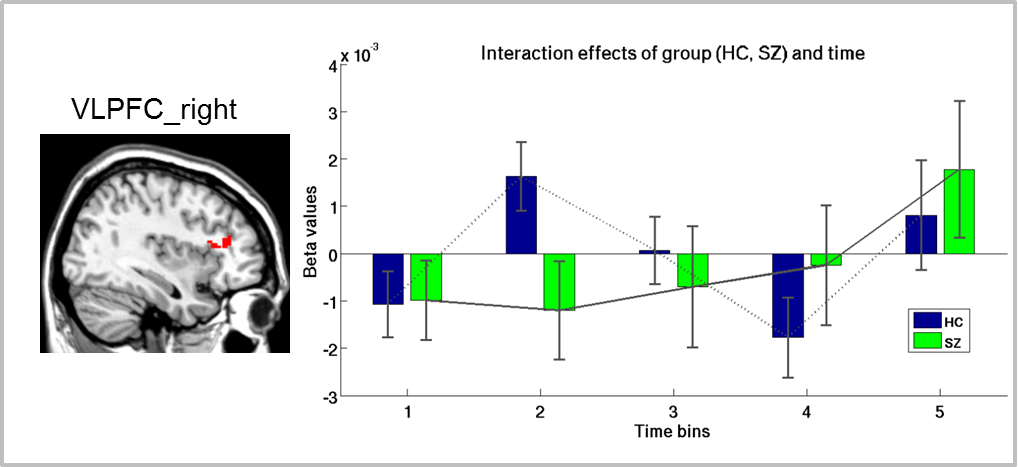


**Supplementary Figure 2** Temporal deviations in patients relative to HC during *reappraise*>*attend negative*. Interaction effects between group and time in SZ and HC groups were observed in the right VLPFC. The line connecting the mean responses in each time bin visualizes the temporal response for each group (HC, dashed light gray; SZ, straight dark gray). Abbreviations: HC=healthy controls; SZ=schizophrenia patients; VLPFC=ventrolateral prefrontal Cortex.


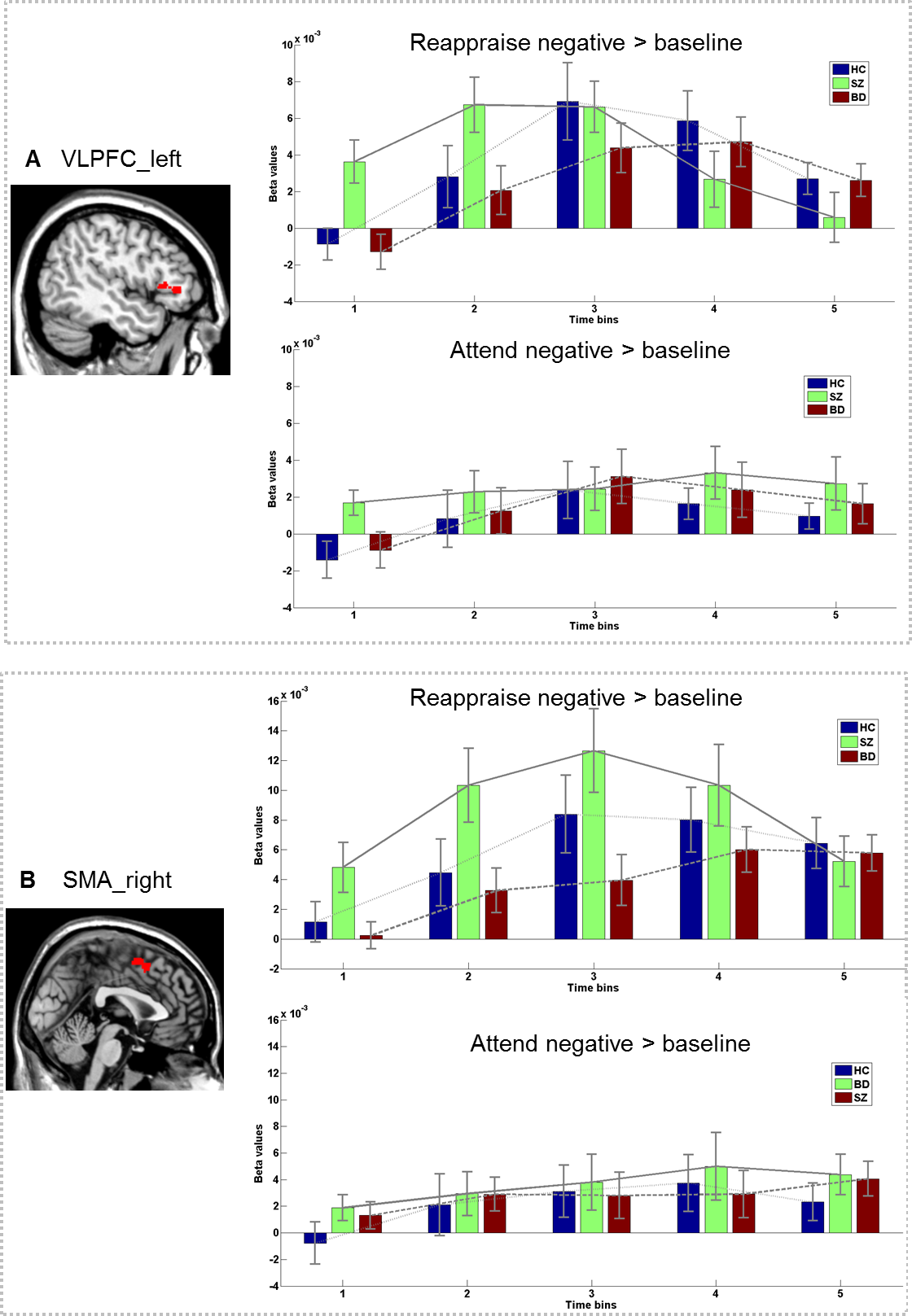


**
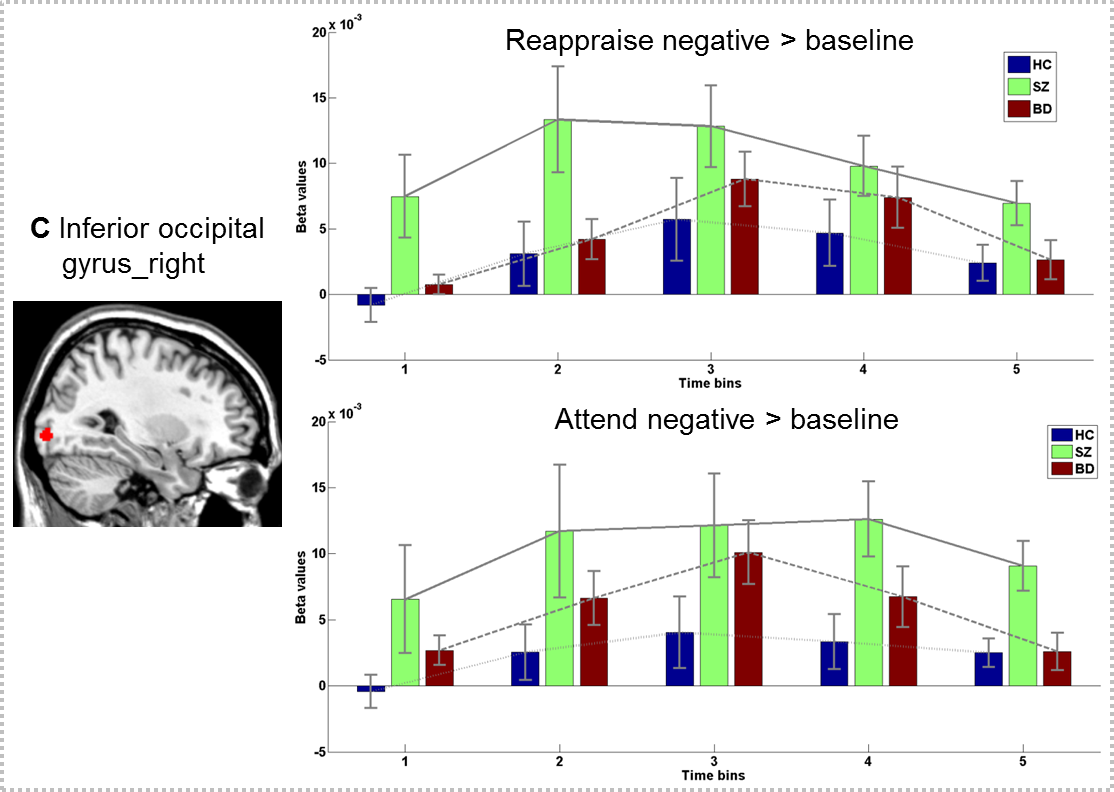
**

**Supplementary Figure 3.** Temporal profiles of activated clusters from reappraisal condition (Reappraise > attend negative) in healthy control, patients with bipolar disorder and schizophrenia at the contrast *reappraise negative > baseline* and *attend negative > baseline*. Effects are observed in the (A) VLPFC, (B) SMA, and (C) inferior occipital gyrus. Time bins start at the presentation of regulation cue (i.e., time bin 1, 0-2 seconds post instruction). The presented time course is averaged over significant voxels. The line connecting the mean responses in each time bin visualizes the temporal response for each group (BD, dashed dark gray; HC, dashed light gray; SZ, straight dark gray). Abbreviations: BD=bipolar disorder; HC=healthy controls; SZ=schizophrenia; SMA=supplementary motor area; VLPFC=ventrolateral prefrontal cortex.


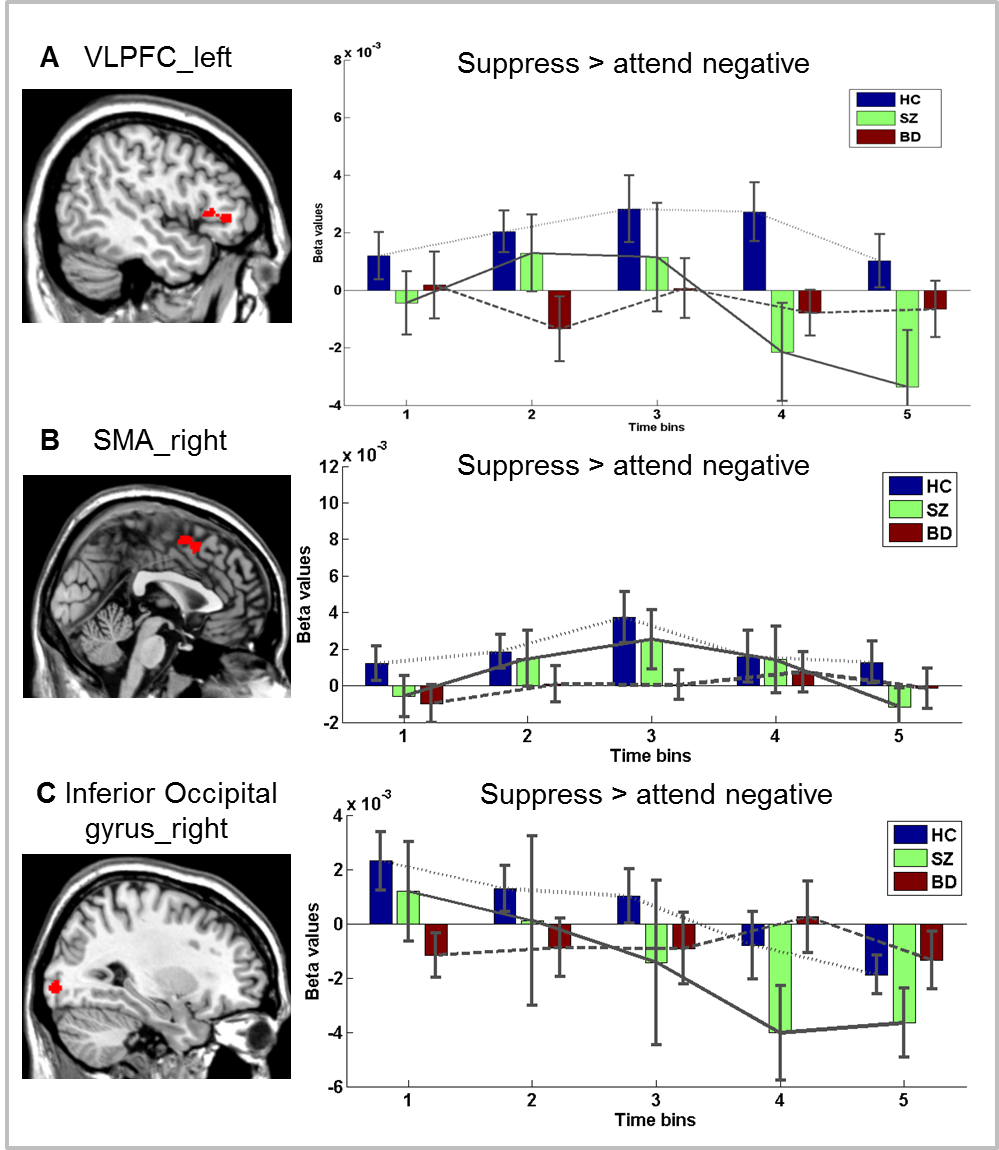


**Supplementary Figure 4.** Effect of *suppress > attend negative* on the areas from main analyses: (A) VLPFC, (B) SMA, and (C) inferior occipital gyrus. Scales were the same as main analyses results. Abbreviations: BD=bipolar disorder; HC=healthy controls; SZ=schizophrenia; SMA=supplementary motor area; VLPFC=ventrolateral prefrontal cortex.


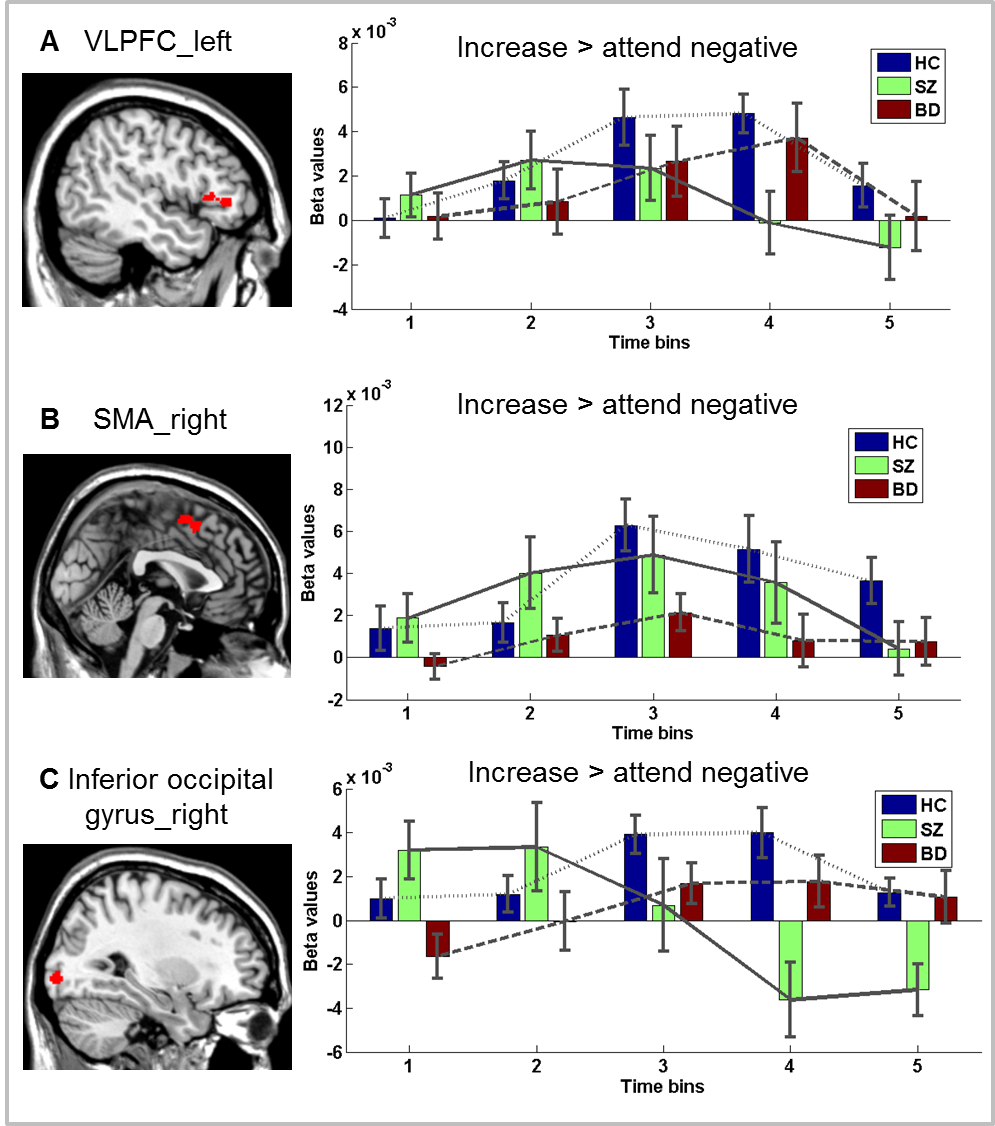


**Supplementary Figure 5.** Effect of *increase > attend negative* on the areas from main analyses: (A) VLPFC, (B) SMA, and (C) inferior occipital gyrus. Scales were the same as main analyses results. Abbreviations: BD=bipolar disorder; HC=healthy controls; SZ=schizophrenia; SMA=supplementary motor area; VLPFC=ventrolateral prefrontal cortex


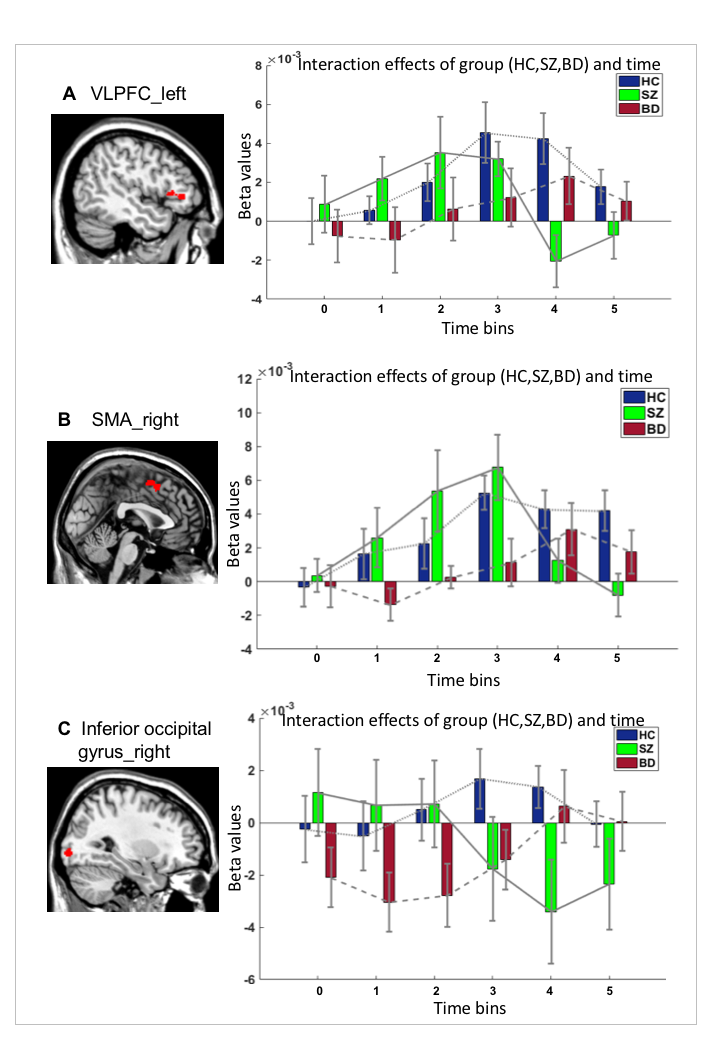


**Supplementary Figure 6.** Temporal profiles reflecting ‘Reappraise > attend negative’ over six time bins in healthy controls, patients with bipolar disorder and patients with schizophrenia in the (A) VLPFC, (B) SMA, and (C) inferior occipital gyrus. Here, time bin 0 reflects the viewing phase, and time bin 1 is the first bin following the cue to either reappraise or attend. The presented time course is derived from averaging over significant voxels resulting from the main analysis. Abbreviations: BD=bipolar disorder; HC=healthy controls; SZ=schizophrenia; SMA=supplementary motor area; VLPFC=ventrolateral prefrontal cortex
